# Hip flexor and ankle dorsiflexor strength associated with gait speed in post-stroke hemiparesis: Cross-sectional study

**DOI:** 10.1016/j.jtumed.2026.05.011

**Published:** 2026-06-04

**Authors:** Vishal Vennu, Emad Moftah, Saad M. Bindawas

**Affiliations:** a Department of Rehabilitation Sciences, College of Applied Medical Sciences, King Saud University, Riyadh, KSA; b Rehabilitation Services Department, King Abdulaziz Medical City, Ministry of the National Guard-Health Affairs, Riyadh, KSA

**Keywords:** بسط الكاحل, سعة المشي, ثني الفخذ, الشلل النصفي بعد السكتة الدماغية, إعادة تأهيل السكتة الدماغية, Ankle dorsiflexion, Gait speed, Hip flexion, Post-stroke hemiparesis, Stroke rehabilitation

## Abstract

**Objectives:**

Lower-limb flexor muscle strength, including hip flexor (HF) and ankle dorsiflexor (AD) strength, is important for limb advancement and walking after stroke. However, their individual and combined associations with gait speed (GS) in adults with post-stroke hemiparesis are not well understood, especially in KSA. Thus, the present study examined the individual and combined associations of HF and AD strength with GS in this population.

**Methods:**

This cross-sectional study included 60 ambulatory adults with post-stroke hemiparesis who were recruited from a tertiary rehabilitation hospital in Riyadh. Isometric HF and AD strength (N·m/kg) were measured using standardized handheld dynamometry. GS (m/s) was assessed using an electronic walkway. Associations were analyzed based on Pearson's correlation coefficients (*r*) and linear regression, both unadjusted and adjusted for age, sex, stroke type, stroke chronicity, hemiparesis side, and body mass index.

**Results:**

HF strength was more strongly correlated with GS (*r* = 0.45; 95% confidence interval [CI], 0.22–0.63; *P* < 0.001) than AD strength (*r* = 0.27; 95% CI, 0.01–0.49; P = 0.041). In the adjusted regression models, HF strength remained significantly associated with GS (β = 0.74; *P* = 0.001), explaining 41% of the variance. AD was not significantly associated with GS when both variables were included. These coefficients were partially adjusted because the models excluded relevant muscle groups, such as knee extensors and ankle plantar flexors.

**Conclusion:**

HF strength was associated with GS in post-stroke hemiparesis, whereas AD strength did not provide additional explanatory value. These findings may support prioritizing proximal lower-limb strengthening during stroke rehabilitation.

## الملخص

**أهداف البحث:**

تُعد قوة العضلات القابضة في الطرف السفلي، بما في ذلك ثني الفخذ (HF) وبسط الكاحل (AD)، ضرورية لحركة الأطراف والمشي بعد السكتة الدماغية. ومع ذلك، فإن ارتباطاتها الفردية والمشتركة بسعة المشي (GS) لدى البالغين المصابين بالشلل النصفي بعد السكتة الدماغية ليست مفهومة تماماً، لا سيما في المملكة العربية السعودية. هدفت هذه الدراسة إلى فحص الارتباطات الفردية والمشتركة لقوة ثني الفخذ وبسط الكاحل مع سعة المشي في هذه الفئة من المرضى.

**طرق البحث:**

شملت هذه الدراسة المقطعية 60 بالغاً من القادرين على المشي المصابين بالشلل النصفي بعد السكتة الدماغية، والذين تم استقطابهم من مستشفى تأهيلي تخصصي في الرياض. تم قياس قوة ثني الفخذ وبسط الكاحل باستخدام مقياس دينامومتر يدوي قياسي، كما تم تقييم سعة المشي (متر/ثانية) باستخدام اختبار المشي لمدة 10 أمتار. واستُخدمت نماذج الانحدار الخطي لتقييم الارتباطات بين قوة العضلات وسعة المشي بعد تعديل المتغيرات الديموغرافية والسريرية.

**النتائج:**

أظهرت النتائج وجود ارتباطات طردية معنوية بين سعة المشي وكل من قوة ثني الفخذ (P = 0.003) وقوة بسط الكاحل (P = 0.041) بشكل فردي. وعند دمج المتغيرين في نموذج واحد، ظلت قوة ثني الفخذ مرتبطة بشكل قوي ومعنوي بسعة المشي (P = 0.002)، بينما تلاشت المعنوية الإحصائية لقوة بسط الكاحل .(P = 0.285) وأشارت النتائج إلى أن قوة ثني الفخذ كانت المتنبئ الأقوى والأكثر استقلالية لسرعة المشي في هذه العينة.

**الاستنتاج:**

ترتبط قوة العضلات القابضة للفخذ والباسطة للكاحل بشكل فردي بسعة المشي لدى المصابين بالشلل النصفي بعد السكتة الدماغية، إلا أن قوة ثني الفخذ تبرز كعامل محدد رئيسي وأكثر أهمية للقدرة على المشي. تشير هذه النتائج إلى ضرورة تركيز برامج إعادة التأهيل بعد السكتة الدماغية في المملكة العربية السعودية على تمارين تقوية ثني الفخذ بشكل خاص لتعزيز استعادة القدرة على المشي وتحسين النتائج الوظيفية للمرضى.

## Introduction

Gait impairment is a primary consequence of stroke-related hemiparesis. Gait speed (GS) is a standard indicator of functional mobility, independence, and rehabilitation outcomes.^1^, ^2^, ^3^, ^4^ Despite its clinical utility, GS represents a measure of the total functional output rather than individual joint mechanics, and thus does not delineate the underlying biomechanical and neuromotor determinants of walking recovery.^5^ Consequently, identifying modifiable physiological contributors to GS remains a priority for optimizing targeted rehabilitation strategies.^6^, ^7^, ^8^, ^9^

Lower-limb flexor musculature, particularly the hip flexors (HF) and ankle dorsiflexors (AD), plays a critical role in limb advancement, step length generation, and foot clearance during gait.^7^^,^^10^ Previous studies have demonstrated associations between lower-limb strength and GS, but most evaluated muscle groups in isolation or as composite indices, limiting insights into their relative contributions after adjustment within a multivariable framework.^8^^,^^11^^,^^12^ Ankle plantar flexors play a primary role in propulsion during gait, so the present study focused specifically on HF and AD strength due to their roles in limb advancement and foot clearance during the swing phase. This targeted approach allowed us to evaluate proximal and distal contributions to limb advancement; however, it did not capture the full spectrum of the biomechanical determinants of GS.

Therefore, this limitation was addressed by directly comparing HF and AD strength within a unified regression framework using standardized dynamometry-derived torque measures. This approach allowed us to delineate their associations with GS while accounting for shared variance and relevant clinical covariates. We hypothesized that both HF and AD strength would be associated with GS, but that their relative contributions would differ after multivariable adjustment.

## Materials and Methods

### Study design and setting

A cross-sectional study was conducted in inpatient and outpatient neurorehabilitation services at a tertiary care hospital in Riyadh, KSA. The institutional review board approved the study and all participants provided written informed consent prior to participation. The procedures adhered to the Declaration of Helsinki, and reporting followed the Strengthening the Reporting of Observational Studies in Epidemiology (STROBE) guidelines.^13^

### Sample size

Sample size was determined by a priori power analysis conducted in G∗Power (version 3.1), informed by effect size estimates from previous studies that examined lower-limb strength and GS relationships in comparable post-stroke populations.^7^ A priori power analysis indicated that 58 participants were required to detect a bivariate correlation of r = 0.35. This calculation assumed 80% power and a two-sided α of 0.05. In total, 60 adults were enrolled to ensure an adequate sample size after accounting for potential attrition and post-screening data exclusion.

### Participants

Adults aged >55 years with a confirmed diagnosis of stroke and resultant hemiparesis with at least 1 week's duration were recruited between May 2017 and August 2018 from the hospital's stroke registry. Eligible participants were required to walk ≥6 m independently, with or without a prescribed assistive device. Both inpatients and outpatients who were actively enrolled in rehabilitation services were considered for inclusion. Data were collected between 2017 and 2018, but the analysis and manuscript preparation incorporated the most recent literature.

Participants were excluded if they presented with any of the following: lower-limb pain exceeding 4 out of 10 on the Numeric Pain Rating Scale and that impaired gait mechanics, including pain attributable to osteoarthritis; moderate or severe lower-limb spasticity, defined as grade 3 or 4 on the Modified Ashworth Scale, affecting the hamstrings, quadriceps, hip adductors, or calf musculature; scissoring gait secondary to hip adductor spasticity; severe pathology of the hip, knee, or ankle joint; a history of significant pre-existing neurological deficits; or cognitive or language impairment that precluded the provision of informed consent or the ability to follow standardized testing instructions. Three experienced physiotherapists performed all assessments following a standardized testing protocol. The assessors received training in the study procedures but formal blinding to the participants’ characteristics was not implemented.

### Measures

Isometric HF and AD strength were measured using a calibrated handheld dynamometer (JTech Commander PowerTrack; JTech Medical, Midvale, UT, USA). For each muscle group, three maximal isometric trials were performed, and the mean value was used for analysis, consistent with dynamometry reliability protocols established for neurological populations.^14^^,^^15^ AD strength was assessed with participants in the supine position, hips and knees in full extension, and with the dynamometer contact pad positioned over the dorsal aspect of the foot proximal to the metatarsal heads. HF strength was assessed with the hip positioned at 90° of flexion. Raw isometric force values in Newtons were converted to joint torque (N·m) by multiplying the recorded force by the perpendicular distance from the dynamometer contact point to the respective joint axis, which were measured individually for each participant. Torque values were normalized to body mass, yielding units of N·m/kg, to facilitate strength comparisons across participants with varying anthropometric characteristics. It has been demonstrated that this normalized torque approach has high intra-rater and inter-rater reliability in post-stroke and broader neurological populations, with reported intraclass correlation coefficients ranging from 0.85 to 0.95.^14^^,^^15^

GS was assessed using a Zebris-Mat pressure-sensitive electronic walkway system (Zebris Medical GmbH, Isny im Allgäu, Germany). Participants walked along a standardized pathway at their self-selected comfortable pace, and temporospatial gait parameters, including walking speed in meters per second, were automatically recorded and exported for analysis. It has been demonstrated that the Zebris-Mat system has high test–retest reliability in older adult populations, with reported within-day and between-day coefficients of variation below 5% and 7%, respectively.^16^^,^^17^

The following demographic and clinical variables were recorded as potential confounders: age, sex, body mass index (BMI; kg/m^2^), stroke chronicity (subacute: less than six months post-stroke; chronic: six months or more), stroke type (ischemic or hemorrhagic/transient ischemic attack), and side of hemiparesis (right or left). All covariate data were extracted from the participants' medical records and verified through structured participant interviews.

### Statistical analysis

We assessed data normality using the Shapiro–Wilk test before selecting descriptive statistics and inferential methods. We expressed continuous variables with normal distributions as the mean ± standard deviation, non-normally distributed continuous variables as the median and interquartile range (IQR), and categorical variables as frequencies with percentages. Bivariate associations between each muscle group and GS were quantified using Pearson's correlation coefficients (*r*), which were reported with 95% confidence intervals (CIs) and two-tailed *P*-values.

To evaluate the individual and joint contributions of HF and AD strength to GS, three sequential sets of linear regression models were constructed. First, unadjusted individual models assessed the association of each muscle group with GS in isolation. Second, an unadjusted combined model included both predictors simultaneously to assess their relative contributions without covariate control. Third, multivariable-adjusted models were constructed in both single- and combined-predictor forms, controlling for age, sex, stroke type, hemiparesis side, stroke chronicity, and BMI. Prior to combined model construction, multicollinearity between HF and AD strength was assessed using the variance inflation factor, where values exceeding 10 were pre-specified as indicative of problematic collinearity. All regression outputs were reported as unstandardized regression coefficients (β) with standard error (SE), *P*-values, and unadjusted or adjusted coefficients of determination (R^2^ or Adjusted R^2^). All statistical analyses were performed using R version 4.2.0 (R Foundation for Statistical Computing, Vienna, Austria). A two-sided *P*-value below 0.05 was considered to indicate a statistically significant difference. No data were missing for any of the primary outcome variables.

## Results

### Participants’ characteristics

**Figure 1:**
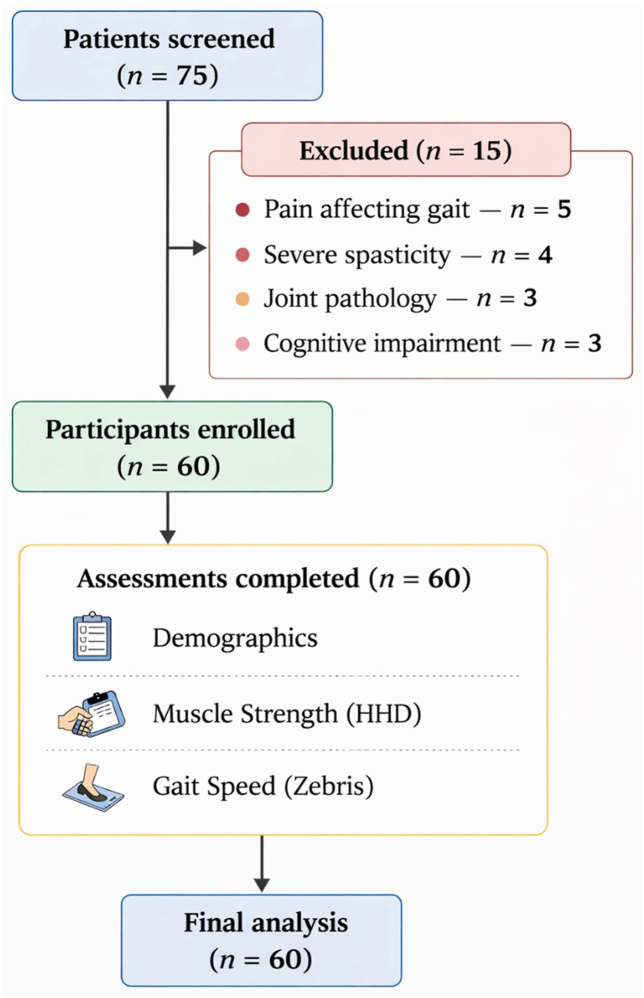
Flow diagram illustrating the recruitment and inclusion of participants for the study.

Among the 75 patients screened, 60 met the eligibility criteria and were included in the analysis (Figure 1). Participants had a median age of 66 years (IQR: 60–72), were predominantly male (63%), with chronic (60%) ischemic stroke (82%), right-sided hemiparesis (62%), and elevated BMI (32.4 ± 6.2 kg/m^2^) (Table 1). The mean normalized torque was higher for HF (0.41 ± 0.23 N m/kg) than AD (0.13 ± 0.08 N m/kg), while the median GS was 0.39 m/s (IQR: 0.28–0.54), reflecting restricted community ambulation.

**Table 1:** Baseline characteristics of study participants (N = 60).

Characteristic	Value
**Demographics**	
Age, median (IQR), years	66 (60–72)
Male sex, n (%)	38 (63)
BMI, mean ± SD, kg/m^2^	32.4 ± 6.2
**Stroke characteristics**	
Time since stroke, median (IQR), months	8.5 (4.0–18.3)
Stroke chronicity, n (%)	
Subacute (<6 months)	24 (40)
Chronic (≥6 months)	36 (60)
Stroke type, n (%)	
Ischemic	49 (82)
Hemorrhagic/TIA	11 (18)
Side of hemiparesis, n (%)	
Right	37 (62)
Left	23 (38)
**Outcome measures**	
AD strength in N·m/kg, mean ± SD	0.13 ± 0.08
HF strength in N·m/kg, mean ± SD	0.41 ± 0.23
Gait speed in m/s, median (IQR)	0.39 (0.28–0.54)

Abbreviations: AD, ankle dorsiflexion; BMI, body mass index; HF, hip flexion; IQR, interquartile range; SD, standard deviation; TIA, transient ischemic attack.

### Associations of HF and AD strength with GS

According to both the adjusted single- and combined-predictor models controlling for age, sex, stroke type, hemiparesis side, stroke chronicity, and BMI, HF strength remained significantly associated with GS (combined model: β = 0.74; SE = 0.20; *P* = 0.001; Adjusted R^2^ = 0.410) (Table 2). HF alone explained approximately 41% of the variance in GS.

**Table 2:** Associations of lower limb muscle strength with gait speed in post-stroke hemiparesis (unadjusted, multiple, and adjusted models).

Predictor	Unadjusted β (SE)	*P*	Multiple β (SE)	*P*	Adjusted β (SE)	*P*
AD	0.65 (0.31)	0.041	−0.48 (0.45)	0.285	0.64 (0.30)	0.038
HF	0.57 (0.15)	0.003	0.76 (0.23)	0.002	0.57 (0.13)	0.001
AD + HF	–	–	–	–	AD: −0.46 (0.40)	0.259
–	–	–	–	–	HF: 0.74 (0.20)	0.001

Abbreviations: β, estimate; AD, ankle dorsiflexion; HF, hip flexion; SE, standard error. Note: Unadjusted models evaluated each muscle group individually, whereas multiple regression models included both AD and HF strength simultaneously. Multivariable regression models were also adjusted for age, sex, stroke type, hemiparesis side, stroke chronicity, and body mass index to control for potential confounding.

**Figure 2:**
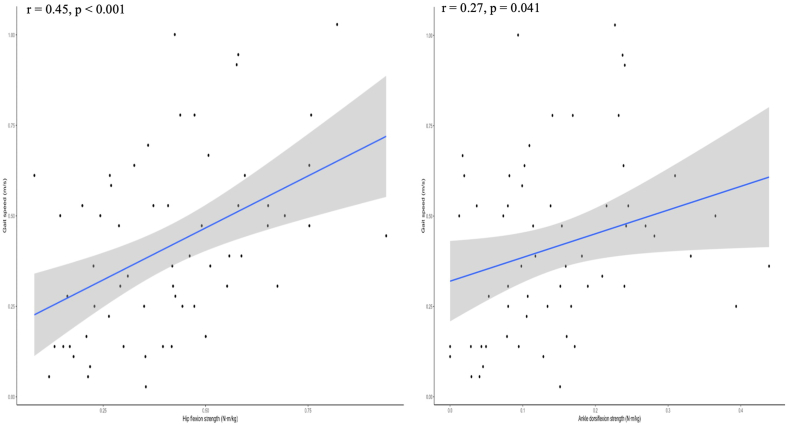
Association between ankle dorsiflexion, hip flexion strengths, and gait speed in adults with post-stroke hemiparesis.

AD strength was correlated with GS according to bivariate analysis (*r* = 0.27; *P* = 0.041) but was not associated with GS in the combined or fully adjusted models (adjusted combined: β = −0.46; *P* = 0.259) (Table 2 and Figure 2). These findings may reflect shared variance with HF strength because AD was only statistically significant when modeled alone with covariate adjustment (β = 0.64; *P* = 0.038).

## Discussion

In this study, the relative contributions of HF and AD strength to GS in adults with post-stroke hemiparesis were examined using a combined regression framework. HF strength had the strongest adjusted association with GS, whereas AD strength had a modest bivariate association that did not persist after adjustment. These findings suggest a statistically prominent role for HF strength but they should be interpreted cautiously and within the broader, multifactorial context of post-stroke locomotor recovery. HF strength was associated with coordinated limb advancement within a constrained neuromuscular system, particularly when distal propulsion was impaired and compensatory motor strategies were required.

HF strength explained a substantial proportion of the variance in GS, but the clinical significance of this association requires careful consideration. In post-stroke populations, the minimal clinically important difference for GS is typically 0.10–0.16 m/s. The observed regression coefficient indicated a statistically significant association but it did not directly show that changes in HF strength would produce clinically meaningful improvements in GS, which further highlights the need for longitudinal and interventional studies to determine whether modifying muscle strength translates to meaningful functional gains.

Stroke-related gait is a complex behavior that involves muscle strength, balance, and proprioception.^2^^,^^4^, ^5^, ^6^ Systematic reviews and meta-analyses consistently demonstrate that GS reflects the integration of impairments across multiple domains rather than the contribution of a single muscle group.^7^^,^^9^^,^^18^ Interventions targeting higher-level motor control, such as dual-task training, further indicate that improvements in mobility and balance are mediated by enhanced cognitive–motor integration rather than isolated strength gains.^19^ HF strength accounts for a substantial proportion of the variance in GS but is not the sole physiological driver. Instead, it functions as an indicator of global corticospinal tract integrity. In support of this interpretation, a previous study demonstrated that global lower-limb motor function assessed by using the Fugl–Meyer Assessment correlated more strongly with GS than isolated hip muscle strength,^20^ suggesting that HF strength may act as a surrogate marker of overall corticospinal tract integrity rather than as an independent mechanical driver of walking performance.

From a biomechanical perspective, HF muscles contribute to limb advancement and step initiation during the swing phase, where these functions are closely linked to trunk control, pelvic stability, and temporal coordination during gait.^21^, ^22^, ^23^ Experimental and observational studies further suggest that GS is influenced by spatiotemporal parameters and asymmetry patterns, which are shaped by coordinated multi-joint interactions rather than isolated muscle output.^5^^,^^6^ In this context, the observed association probably reflects the integrated neuromuscular capacity rather than isolated segmental strength. Post-stroke individuals adopt compensatory strategies to maintain forward progression when distal function fails. These strategies include altered pelvic kinematics and proximal muscle over-recruitment.^21^^,^^23^

Recent research has demonstrated the co-primary role of distal musculature, particularly the ankle plantar flexors, in determining gait performance. Distal propulsion generated during late stance is associated with walking speed and endurance because it governs the trailing limb angle and mechanical energy available to initiate limb advancement.^24^^,^^25^ Therefore, impairments in plantarflexion can constrain proximal kinematics as a downstream consequence, limiting effective swing initiation despite adequate HF capacity.^24^^,^^25^ This distal-to-proximal mechanistic chain is further supported by experimental demonstrations that targeted assistance of paretic ankle function using soft robotic exosuits without direct hip intervention result in clinically meaningful improvements in GS and walking distance, accompanied by secondary normalization of hip and knee kinematics.^1^^,^^26^^,^^27^ These findings suggest that the distal propulsive capacity may be an important contributing factor and that improvements in proximal motion may reflect system-level adaptation rather than direct enhancement of HF function.

Similarly, the role of AD strength in the present study warrants nuanced interpretation. AD strength did not remain significant in the multivariable models but dorsiflexor function remains essential for foot clearance, preventing foot drop, and adaptability to environmental demands such as obstacle negotiation.^28^ Its attenuated contribution may reflect shared variance with HF strength, overlap with unmeasured constructs, such as balance and motor control, or inherent limitations in isometric strength assessment. Previous evidence indicates that balance impairments and asymmetry are strongly associated with gait dysfunction in stroke populations,^4^^,^^6^ and that distal muscle performance contributes to gait safety and efficiency even when not predictive.^7^^,^^11^

Available evidence demonstrates the interdependence of proximal and distal muscle groups in post-stroke gait. HF strength functions as an index of global neuromuscular recovery rather than a singular physiological determinant. By contrast, distal propulsion appears to be more directly associated with GS, highlighting the need to interpret proximal strength findings within a system-level framework. Therefore, a critical synthesis of supporting and contrasting evidence suggests that the observed associations are more consistent with integrated system behavior than isolated causal mechanisms.

Compared with previous research,^7^^,^^8^^,^^11^^,^^14^^,^^15^^,^^22^ the present study contributes by directly evaluating HF and AD strength within a combined regression framework using standardized dynamometry, offering a reproducible and clinically accessible approach to strength assessment.^21^^,^^23^^,^^28^^,^^29^ The reliability of dynamometry and gait measurement approaches is well established in both clinical and research settings.^14^, ^15^, ^16^, ^17^ However, these findings must be interpreted in light of important methodological constraints. Several key determinants of gait performance were not measured, including balance, spasticity severity, motor control, trunk stability, proprioception, and cardiovascular fitness, thereby introducing the possibility of residual confounding. In addition, the cross-sectional design precludes causal inference, and the single-center sample may limit generalizability.

Clinically, these results suggest that the assessment and strengthening of HF muscles may be a useful component of post-stroke rehabilitation,^22^^,^^30^ interventions should not disproportionately prioritize proximal musculature. Instead, rehabilitation strategies should remain comprehensive, incorporating distal propulsion training, balance and postural control, neuromuscular re-education, and task-specific gait practice.^14^^,^^15^^,^^31^ Distal muscles, including ankle plantar flexors and dorsiflexors, drive gait efficiency and adaptability despite the lack of statistical significance in this model. Framing rehabilitation within this integrated perspective may enhance the translation of findings into effective, individualized clinical practice.^18^^,^^32^, ^33^, ^34^, ^35^, ^36^ Broader rehabilitation evidence also demonstrates that multidimensional interventions, rather than isolated impairments, drive improvements in mobility and quality of life after stroke.^2^^,^^3^^,^^18^^,^^19^^,^^32^, ^33^, ^34^, ^35^, ^36^ Furthermore, given the rising burden of stroke and increasing demand for rehabilitation services globally and regionally, optimizing efficient, evidence-based gait rehabilitation strategies remains a critical priority.^37^

## Limitations

This study had several limitations that should be considered when interpreting the findings. First, the cross-sectional design precludes any inference regarding causality or directionality. HF strength was associated with GS but it is unclear whether greater strength facilitates faster walking, whether higher-functioning individuals develop greater strength through activity, or whether both are driven by underlying neurological recovery.

Second, several covariates were included, but key determinants of gait performance were not assessed, such as balance, spasticity severity within the inclusion range, motor control, proprioception, trunk stability, and cardiovascular fitness. The omission of these variables introduces potential residual confounding and may influence the observed associations, potentially inflating the apparent contribution of HF strength.

Third, the regression models included only HF and AD strength, and did not account for other relevant muscle groups, such as knee extensors and ankle plantarflexors. Consequently, the reported coefficients represent partially adjusted estimates and may be subject to omitted variable bias.^2^^,^^3^ This limitation may have led to model misspecification, potentially overestimating the relative importance of HF strength but underrepresenting distal contributions.

Fourth, the use of handheld dynamometry is reliable, but captures isometric strength under controlled conditions and may not reflect dynamic muscle performance during walking, which involves coordinated, velocity-dependent muscle activity.^29^^,^^30^ In addition, the lack of assessor blinding may have introduced measurement bias.

Fifth, the participants were recruited from a single tertiary rehabilitation center, which may limit any generalizability to broader stroke populations. Finally, the sample size was adequate for primary analyses but it may not have been sufficient to detect smaller associations, particularly for AD strength within multivariable models. Collectively, these limitations suggest that the predictive value of HF strength may be overestimated, whereas the contribution of distal musculature may be underestimated.

## Conclusion

In adults with post-stroke hemiparesis, HF strength had the strongest association with GS, whereas AD strength had only a modest bivariate relationship and no significant association after adjustment. However, these findings should not be interpreted as evidence of a causal relationship. HF strength probably reflects broader neuromuscular function, including global motor recovery and coordination. Previous evidence also highlights the role of distal musculature, particularly ankle plantar flexors, in gait performance. Clinically, these results support a comprehensive rehabilitation approach that targets both proximal and distal muscle groups alongside balance, neuromuscular control, and task-specific gait training. Longitudinal and interventional studies are required to determine the temporal and mechanistic contributions of proximal and distal muscle groups to gait performance.

## Ethical approval

The institutional review board of King Abdullah International Medical Research Center approved this study (approval number: SP17/067/R) on 23rd April 2018. All participants provided written informed consent before enrollment.

## Data availability statement

The data used or analyzed for this study are not publicly available due to privacy restrictions.

## Authors contributions

Conceptualization: EM, VV, and SMB; Methodology: EM and VV; Data analysis: EM and VV; Writing original draft: VV and EM; Writing – review and editing: SMB; Supervision: SMB. All authors have critically reviewed and approved the final draft and are responsible for the content and similarity index of the manuscript.

## Source of funding

This work was supported by the Ongoing Research Funding program (funding number: ORF-2026-1094), King Saud University, Riyadh, KSA.

## Conflict of interest

The authors have no conflicts of interest to declare.
